# Complete Genome Sequence of Polyvinyl Alcohol-Degrading Strain *Sphingopyxis* sp. 113P3 (NBRC 111507)

**DOI:** 10.1128/genomeA.01169-15

**Published:** 2015-10-15

**Authors:** Yoshiyuki Ohtsubo, Yuji Nagata, Mitsuru Numata, Kieko Tsuchikane, Akira Hosoyama, Atsushi Yamazoe, Masataka Tsuda, Nobuyuki Fujita, Fusako Kawai

**Affiliations:** a Department of Environmental Life Sciences, Graduate School of Life Sciences, Tohoku University, Sendai, Japan; b Biological Resource Center, National Institute of Technology and Evaluation, Tokyo, Japan; c Center for Fiber and Textile Science, Kyoto Institute of Technology, Kyoto, Japan

## Abstract

Strain 113P3 was isolated from activated sludge and identified as a polyvinyl alcohol (PVA)-degrading *Pseudomonas* species; it was later reidentified as *Sphingopyxis* species. Only three genes are directly relevant to the metabolism of PVA and comprise the *pva* operon, which was deposited as accession no. AB190228. Here, we report the complete genome sequence of strain 113P3, which has been conserved as a stock culture (NBRC 111507) at the Biological Resource Center, National Institute of Technology and Evaluation (NITE) (Tokyo, Japan). The genome of strain 113P3 is composed of a 4.4-Mb circular chromosome and a 243-kb plasmid. The whole finishing was conducted *in silico* except for four PCRs. The sequence corresponding to AB190288 exists on the chromosome.

## GENOME ANNOUNCEMENT

Strain 113P3 was isolated as the polyvinyl alcohol (PVA)-utilizing *Pseudomonas* sp. 113P3 (1, 2), which was reidentified as *Sphingopyxis* sp. 113P3 (3–5) based on proposals by Yabuuchi et al. (6, 7) and Takeuchi et al. (8). PVA is depolymerized by PVA dehydrogenase (PVA-DH) (2, 4), linked with cytochrome *c* (5) and oxidized-PVA hydrolase (OPH) (3). All together, three genes encoding PVA-DH, OPH, and cytochrome *c* comprise the *pva* operon located on a megaplasmid (9, 10).

The strain 113P3 genome was sequenced using 454 GS-FLX Titanium (Roche) and HiSeq and MiSeq systems (Illumina). A fragment library was constructed for the 454 GS-FLX sequencing, and the obtained reads were subjected to 21-mer based trimming by ShortReadManager (SRM), in which 21-mers occurring only once were excluded. For the Illumina HiSeq sequencing, a mate-pair (MP) library was constructed and sequenced for 151 bp apiece from both ends to obtain 69.7 M reads. The mate pairs were extracted and trimmed by SRM, in which 21-mers occurring more than three times were regarded as valid. For Illumina MiSeq sequencing, a paired-end (PE) library was constructed and sequenced for 301 bp apiece from both ends. A perl script was used to merge pair reads so that two reads overlap completely for >30 bases. The merged reads were then subjected to SRM trimming. We used Newbler version 2.8 to assemble those reads (we used 53,640 reads from the GS-FLX sequencing [22.2 Mb], 110,524 merged PE reads [43.2 Mb], and 3,692,446 MP reads [332 Mb]), obtaining 239 contigs and nine scaffolds. The finishing was facilitated using GenoFinisher and AceFileViewer (11). Scaffold adjacencies were determined by GenoFinisher, except those among 4 scaffold ends, for which PCR experiments were conducted. The DNA sequences of all gaps were determined by GenoFinisher. The DNA sequence of the repeat-induced gaps was determined by AceFileViewer. For gaps from a lack of reads, the wealth of MP reads was searched using SRM to find reads that fit the gaps. For the slightly overlapping gaps, the distance distribution of mate pairs between gaps was compared with that of mate pairs that mapped to the largest contig. The finished sequence was confirmed by FinishChecker, annotated by the NCBI Prokaryotic Genomes Automatic Annotation Pipeline (PGAP), and curated using GenomeMatcher (12). While referring to annotation data obtained from the Microbial Genome Annotation Pipeline (http://www.migap.org/), we corrected start codon positions and added genes that were missing in the PGAP annotation.

The complete sequence of the strain 113P3 genome comprised one circular chromosome of 5,174,928 bp and one plasmid of 243,437 bp. A stretch of DNA sequence including the *pva* operon (accession no. AB190288) (9) was found on the chromosome.

### Nucleotide sequence accession numbers.

The genome sequence of *Sphingopyxis* sp. 113P3 has been deposited in the NCBI under the accession numbers CP009452 to CP009453.
